# Cognitive Training to Enhance Aphasia Therapy (Co-TrEAT): A Feasibility Study

**DOI:** 10.3389/fresc.2022.815780

**Published:** 2022-04-05

**Authors:** Tijana Simic, Laura Laird, Nadia Brisson, Kathy Moretti, Jean-Luc Théorêt, Sandra E. Black, Gail A. Eskes, Carol Leonard, Elizabeth Rochon

**Affiliations:** ^1^Centre de Recherche de l'Institut Universitaire de Gériatrie de Montréal (CRIUGM), Montreal, QC, Canada; ^2^Heart and Stroke Foundation Canadian Partnership for Stroke Recovery, Ottawa, ON, Canada; ^3^Department of Speech-Language Pathology, University of Toronto, Toronto, ON, Canada; ^4^Rehabilitation Sciences Institute, University of Toronto, Toronto, ON, Canada; ^5^School of Rehabilitation Sciences, University of Ottawa, Ottawa, ON, Canada; ^6^Division of Neurology, Department of Medicine, Sunnybrook Health Sciences Centre, University of Toronto, Toronto, ON, Canada; ^7^KITE Research Institute, Toronto Rehab, University Health Network, Toronto, ON, Canada; ^8^Departments of Psychiatry and Psychology and Neuroscience, Dalhousie University, Halifax, NS, Canada

**Keywords:** aphasia, working memory, rehabilitation, multi-modal therapy, anomia

## Abstract

Persons with aphasia (PWA) often have deficits in cognitive domains such as working memory (WM), which are negatively correlated with recovery, and studies have targeted WM deficits in aphasia therapy. To our knowledge, however, no study has examined the efficacy of multi-modal training which includes both WM training and targeted language therapy. This pilot project examined the feasibility and preliminary efficacy of combining WM training and naming therapy to treat post-stroke PWA. Chronic PWA were randomly assigned to either the a) Phonological Components Analysis (PCA) and WM intervention (WMI) condition (i.e., a computerized adaptive dual n-back task), or b) PCA and active control condition (WMC). Participants received face-to-face PCA therapy 3 times/week for 5 weeks, and simultaneously engaged in WM training or the active control condition five times/week, independently at home. Six PWA were enrolled, 3 in each condition. Feasibility metrics were excellent for protocol compliance, retention rate and lack of adverse events. Recruitment was less successful, with insufficient participants for group analyses. Participants in the WMI (but not the WMC) condition demonstrated a clinically significant (i.e., > 5 points) improvement on the Western Aphasia Battery- Aphasia Quotient (WAB-R AQ) and Boston Naming Test after therapy. Given the small sample size, the performance of two individuals, matched on age, education, naming accuracy pre-treatment, WAB-R AQ and WM abilities was compared. Participant WMI-3 demonstrated a notable increase in WM training performance over the course of therapy; WMC-2 was the matched control. After therapy, WMI-3's naming accuracy for the treated words improved from 30 to 90% (compared to 30–50% for WMC-2) with a 7-point WAB-R AQ increase (compared to 3 for WMC-2). Improvements were also found for WMI-3 but not for WMC-2 on ratings of communicative effectiveness, confidence and some conversation parameters in discourse. This feasibility study demonstrated excellent results for most aspects of Co-TrEAT. Recruitment rate, hampered by limited resources, must be addressed in future trials; remotely delivered aphasia therapy may be a possible solution. Although no firm conclusions can be drawn, the case studies suggest that WM training has the potential to improve language and communication outcomes when combined with aphasia therapy.

## Introduction

Approximately 40% of individuals who survive a stroke will present with aphasia—the inability to produce and/or understand language (1). Aphasia is a “complex clinical entity” (2) that can manifest in a variety of communication impairments, including difficulty producing words and sentences and in understanding spoken and/or written language. A study by Lam and Wodchis (3) found that, out of 60 medical diagnoses and 15 health conditions, aphasia has the largest negative effect on health-related quality of life (QoL), ahead of diagnoses such as cancer and Alzheimer's disease. Indeed, even when physical abilities, well-being and social support are comparable to stroke patients without aphasia, individuals with aphasia engage in fewer extended activities of daily living and report diminished QoL (4). Thus, there is a clear imperative for aphasia to be a focus of investigation for improved optimization of care and improved outcomes in stroke.

There is a large and growing body of literature demonstrating the efficacy of treatments for communication impairments associated with aphasia in the acute and chronic stages post-stroke [e.g., (2, 5–11)]. Brady et al.'s (6) Cochrane review demonstrates that speech therapy induces greater language improvements compared to no therapy, and that group, one-on-one, computer- and volunteer-facilitated treatments appear to be equally effective in improving language outcomes. However, Brady et al. (6) caution that although speech-language therapy improves functional communication (i.e., communication in day-to-day contexts), the benefits do not necessarily hold over time and further research on long-term efficacy is required. In addition, treatment-related benefits are not always seen for specific language deficits (such as naming difficulties). These apparently nonspecific benefits may be due to the lack of targeted treatments in their review. For example, several of the negative studies [e.g., (12, 13)] which had naming as an outcome did not specifically treat naming. Of the studies that did treat naming [e.g., (14)], the outcome measure did not include the treated words. Importantly, studies evaluating the efficacy of therapy specifically aimed at the treatment of naming deficits show robust short- and long-term improvements in naming of the treated words, for most (but not all) individuals who are treated [e.g., (10, 15–18)].

### Phonological Components Analysis Naming Therapy

A well-known targeted treatment for naming is the Phonological Components Analysis (PCA) protocol which uses guided phonological and orthographic cueing to stimulate naming (19, 20). PCA has been shown to be efficacious, significantly improving the short- and long-term naming accuracy of treated words (19, 21–23), as well as the long-term naming accuracy of untreated words (23, 24). Studies have considered the mechanisms of PCA treatment efficacy within the context of the Interactive Activation (IA) model of word retrieval (25–27). Namely, the IA model proposes that word retrieval occurs through feedforward and feedback spreading activation across a network of nodes, organized into three layers of representations: semantic, lexical and phonological. Evidence suggests that PCA therapy may strengthen lexical-phonological connections (23), and/or increase access to semantic network nodes via spreading activation across all levels of representation in the word retrieval network (22, 28). Importantly, despite the overall efficacy of PCA therapy, individual recovery patterns vary and not all who undergo therapy show significant improvements (as is generally the case for most anomia treatments; (15)).

### Non-linguistic Cognitive Abilities in Aphasia Rehabilitation

Increasingly, studies are demonstrating that residual non-linguistic cognitive abilities may play an important role in rehabilitation after acquired brain injury in general (29–31) and can be key predictors of successful language recovery in particular [e.g., (32–35)]. This supports the notion, proposed in reviews of the literature (36, 37), that aphasia rehabilitation must focus not only on content (language representations) but also on process (non-linguistic cognitive structures that support the use of these language representations). Working memory (WM) is one such cognitive process, which is of primary interest in the present study.

WM has been conceptualized by Baddeley (38, 39) as a multi-component system, containing domain-specific buffers for maintenance of verbal (phonological loop) and visuo-spatial (visual sketchpad) representations, together with an episodic buffer for access to long-term storage; the model also includes a domain-general central executive for updating and controlling the contents and efficiency of active buffers (38). Individuals with aphasia have been found to have both verbal [e.g., (40)] and nonverbal [e.g., (36, 41, 42)] WM deficits [e.g., (43)]. In addition, studies have found that WM capacity is significantly associated with naming therapy outcomes, predicting the extent of recovery or response to rehabilitation (34, 44–46). Within the context of the IA model of word retrieval, there is evidence supporting the importance of WM in maintaining linguistic representations at the lexical and phonological access stages of word retrieval, in order for correct naming to occur [(47); see also (48)].

### WM Training in Aphasia

The evidence suggests that treating WM deficits in aphasia may benefit language and/or communication outcomes. For example, one study (49) compared the effects of a WM training program vs. routine speech-language therapy, on memory and language functioning in individuals with post-stroke Broca's aphasia. WM training consisted of category and digit memory span tasks (backwards and forwards) of varying difficulty levels, as well as a paced auditory serial addition task (i.e., adding the last two numbers heard in a continuous list). Compared to routine speech-language therapy (i.e., the control group), individuals in the WM training group showed significant improvements in both trained and untrained WM tasks, as well as the speech fluency, auditory comprehension, naming and repetition subtests of the Persian version of the Western Aphasia Battery (P-WAB-1; (50)). In another study, combined intermittent theta-burst stimulation and computerized WM training were administered to an individual with post-stroke nonfluent aphasia over 10 consecutive daily sessions (51). WM training consisted of computerized span- and mental manipulation tasks of increasing difficulty. Significant improvements were seen on a measure of nonverbal intelligence, and a trend toward improvement was noted on receptive and expressive language tasks (auditory comprehension, following commands, naming, reading). In addition, studies have shown improvements after WM training (Attention Process Training, delayed repetition) on measures of reading comprehension (52) and repetition (53), respectively.

Recently, Zakariás et al. (54) conducted a systematic review of short-term memory (STM)/WM treatments in aphasia. In this study, STM was defined as the temporary maintenance and retrieval of information, whereas WM was defined as the maintenance and mental manipulation of information. Of 17 eligible studies, nine trained STM using repetition and/or recognition tasks, whereas eight trained WM (using e.g., n-back and mental arithmetic tasks). Improvements in STM were noted in 85% of studies training STM, and improvements in WM were noted in 82% of studies training WM. Additionally, improvements in sentence comprehension after training were reported in seven out of nine studies that included this outcome measure. However, the authors caution that the current evidence base remains unclear on the mechanisms underlying the relationship between WM training and language outcomes. In addition, the studies in this review did not administer combined WM training and language therapy; to date, there is limited evidence on the efficacy of such a combined, multi-modal treatment approach.

### The N-Back Task

While a number of tasks can be used to measure and train WM, the n-back task has many positive features for persons with aphasia (55). In the n-back task, a stream of information is monitored with the goal of deciding whether the current item matches an item that was “n” number of trials ago in the sequence. Thus, this task requires both maintenance and updating of information with each trial. Neuroimaging studies indicate this task activates a bilateral fronto-parietal network that overlaps with language networks (56, 57). In addition, the task can be varied parametrically, depending upon the “n” involved and thus difficulty can be individually adjusted. Finally, the task requires a simple recognition response and can present a variety of stimuli, from letters, words, pictures to spatial locations, making it easier to differentiate linguistic and non-linguistic deficits. In addition, Mayer and Murray (55) tested the n-back across different stimuli and WM loads (from 0 to 2-back) and found the n-back was reliable and sensitive to WM deficits in people with aphasia compared to controls.

Adaptive WM training using the n-back has also been found to improve trained and untrained WM tasks and, in at least some studies, generalize to other cognitive functions (e.g., Raven's Progressive Matrices, which measures general non-verbal intelligence and abstract reasoning) in both young and older healthy individuals and patient populations [(58–63)]. Importantly, in a multiple-baseline study (64), three participants with post-stroke aphasia received computerized WM training, which consisted of practice on an n-back task (either with pictures or spoken words) four times per week, for 4 weeks. Post-training, all participants showed some improvement in sentence comprehension and everyday memory activities; two participants additionally demonstrated improvements in functional communication (i.e., assessor ratings of understandability and intelligibility of spoken messages on familiar everyday topics). As such, the n-back task provides a promising method for WM training in aphasia to combine with naming therapy in a multi-modal approach to aphasia therapy.

### Summary and Objectives

In sum, although treatments for post-stroke naming deficits, such as the PCA therapy, have been shown to be efficacious, individual patterns of recovery can be highly variable, and the mechanisms underlying treatment-induced recovery remain somewhat unclear. It has been suggested that non-linguistic cognitive functions, and specifically WM, may play an important role in supporting language recovery. Indeed, WM has been identified as an important factor in supporting lexical retrieval and treatment success for anomia specifically, and studies that have administered WM training to individuals with post-stroke aphasia have reported improved language outcomes, particularly in auditory comprehension and functional communication measures. To our knowledge, however, no study to date has examined the efficacy of multi-modal rehabilitation which includes simultaneous administration of both WM training and a targeted anomia treatment protocol. The present investigation aimed to explore the feasibility and added benefit in communication outcomes, of combining WM training with the PCA naming therapy.

The primary objective of this pilot study was to examine the feasibility (i.e., practicality and acceptability) of combining two established and manualized treatment protocols—one targeting naming deficits in aphasia (i.e., PCA; (19)) and the other targeting WM (i.e., the *N-Igma* WM training task, described below; (65)). In addition, we aimed to examine the added benefit (i.e., preliminary efficacy) of combining naming therapy with WM training to treat individuals with post-stroke aphasia.

## Methods

### Participants

Ethical approval for the present study was granted by the Research Ethics Boards (REBs) of the University of Toronto and the Aphasia Institute, and ethical approval was also obtained from the March of Dimes Aphasia and Communication Disabilities Program REB. Participants were recruited from these referral sites in the Greater Toronto Area. Informed consent was obtained using both written and pictorial materials and supported communication strategies (66, 67). The following inclusion criteria applied to all participants: (a) history of a single left-hemisphere unilateral stroke, (b) in the chronic stage of recovery (i.e., at least 6 months post-onset), (c) presence of aphasia with anomia (i.e., 10–75% accuracy on the Boston Naming Test-BNT; (68)), (d) normal or corrected-to-normal vision and hearing, (e) right-handed, (f) primarily English-speaking, and (g) with computer and Internet access. Participants were excluded from the study if they: (a) were actively engaged in speech therapy at the time of recruitment, (b) presented with severe comprehension deficits (based on WAB-R auditory comprehension scores), (c) had a known history of drug and/or alcohol abuse, and (d) had a known history of major psychiatric and/or neurological illness.

Using guidelines proposed by Bowen et al. (69), the present study measured the feasibility of a combined WM and anomia intervention according to the following areas of focus: practicality (i.e., the extent to which an intervention can be delivered when resources are constrained), acceptability (i.e., how participants react to the intervention), and limited- or preliminary-efficacy testing (i.e., testing the intervention in a convenience sample, with limited statistical power). Thus, a convenience sample was recruited: with consent, participants' files were reviewed, and participants were screened for eligibility according to the inclusion criteria stated above. Eligible participants were enrolled, and randomly assigned to one of two conditions: PCA and WM control (WMC) or PCA and WM intervention (WMI). Participants were blind to condition. In the WMC condition, participants were administered the PCA treatment for anomia, in combination with an active control task (i.e., a matched computer activity that did not require working memory since the task remained stable at span size 1; described below). In the WMI condition, participants were administered PCA treatment in combination with WM training using the computerized adapted dual n-back *N-Igma* task (to be described in detail below).

### PCA Treatment for Anomia

Prior to therapy, participants underwent baseline testing, whereby naming performance on a battery of 198 colored photographs of nouns was assessed on three separate occasions (presentation order was randomized at each administration). Words named incorrectly on at least two of the baseline sessions were pooled and considered to be potential treatment targets. Two lists (30 words each) were created from this pool of words: the treated list, which was targeted in therapy, and the untreated list, which served as a within-participant control. Treated and untreated lists were matched as closely as possible on the variables of semantic category, word frequency and number of syllables. The list of 30 words was then treated using the PCA therapy approach, approximately 1.5 h per day, 3 days a week for 5 weeks. Therapy was administered by a trained research assistant either at the University of Toronto, or in the participants' homes. Briefly, in PCA naming therapy, participants are presented with a picture of the target word and asked to name it. They are given feedback, and regardless of their ability to name the target, they are asked to identify five phonological components related to the target word (e.g., rhymes with? number of syllables?), guided by the use of a chart (for a detailed protocol description please see (19)).

### WM Training

We trained WM in individuals with aphasia using a computerized adaptive dual n-back task called *N-Igma* (65). Briefly, the *N-Igma* task requires participants to monitor two streams of auditory stimuli (e.g., aurally presented letters, numbers or animal sounds) and visual stimuli (e.g., pictures that varied in location of stimuli, or different landscapes) to indicate whether the current stimulus matches the one presented “n” trials ago. The “n” started at 1 and increased adaptively as performance for both streams reached 90% correct over a block. Accuracy and reaction time were collected for each trial and stored on a secure, university-based server. Stimuli were changed and training level reset to *n* = 1 after every 5 days of training to increase interest and prevent development of stimulus-specific strategies.

In the WMI condition, the n-back task was adaptive, and increased (e.g., from 1- to 2-back, etc.) as participants progressed through the task. In the WMC condition, we employed a non-adaptive dual n-back task as the active control, to match all other aspects of the training program, without the working memory component. Thus, participants remained at 1-back (a simple short term recognition task) and also were encouraged to improve speed and accuracy throughout the training. After an initial practice week in which single stream n-back tasks were practiced, participants engaged with the dual stream *N-Igma* task (in either the WMI or WMC active control conditions) 30 min a day, 5 days a week, for 5 weeks (i.e., simultaneously with, and throughout the duration of the PCA therapy). WM training (or active control) was completed on the participants' personal computers, independently at home. Weekly check-ins were conducted with participants to maintain motivation, and to problem-solve any issues in WM training and active control conditions.

### Feasibility: Practicality and Acceptability

We collected data on the practicality and acceptability (69) of administering anomia and WM treatments simultaneously. To assess practicality, the following metrics were tracked, in line with previously published work in individuals with post-stroke aphasia (70): (a) ease of recruitment (success in reaching recruitment targets, number of eligible patients enrolled); (b) compliance (number of participants who completed at least 80% of each of the PCA and WM training sessions); (c) retention rate (number of participants engaged in combined therapy at discharge/total number of participants enrolled); (d) protocol deviations (unforeseen changes from the combined therapy protocol) which were noted weekly by the project coordinator. For the purposes of this pilot study, our initial recruitment target was set at 20 participants (ten in each condition). To assess acceptability, participants completed the System Usability Scale (SUS; (71, 72)), evaluating the usability of the *N-Igma* computerized WM training platform. The SUS comprises ten items evaluating usability characteristics (e.g., satisfaction, ease of use), and rated on a 5-point Likert rating scale, which generates a usability score out of 100 (for scoring procedures, see (71)). The SUS has been used in previous work with the stroke population (65) and has been adapted for use with individuals with aphasia by our group (73). Given the small sample size, descriptive statistics were used to analyze feasibility metrics.

### Feasibility: Preliminary Efficacy and Communication Outcomes

Outcomes related to treatment efficacy were collected pre- and post-training and at 1-month follow-up and included: (a) naming accuracy of the treated and matched untreated words, (b) performance on the Western Aphasia Battery-Revised (WAB-R; (74)), (c) performance on the BNT (68); (d) ratings of functional communication ability and communication confidence, measured, respectively, by the Communication Effectiveness Index (CETI; (75)), and Communication Confidence Rating Scale for Aphasia (CCRSA; (76)), and (e) discourse, using the Discourse Comprehension Test (DCT; (77)). In addition, a 10-min conversation sample with a family member or friend was obtained. Speech samples were examined for changes that have been linked to WM function in discourse, such as coherence and topic maintenance (78). Although improvements in language were of primary interest in the present study, we also tracked changes in WM capacity on both the Wechsler Memory Scale digit span task (79) and the Corsi-block tapping visual span task (80, 81), with a focus on the backward span in each task, given its close association with WM capacity. Statistical analyses for each outcome measure are described below. Post-treatment outcomes were collected and scored by a research associate who did not administer treatment.

Naming of the treated and untreated words was scored as follows: words named correctly within 10 s of stimulus presentation (including self-corrections) were given a score of 1, and words named incorrectly (including paraphasias of all types), or words named beyond the 10 s time limit, were given a score of 0. Treatment-induced changes in naming accuracy of the treated and untreated words (outcome a above) were analyzed using the Weighted Statistics (WEST) approach (82). This approach overcomes problems of autocorrelation and is suitable for evaluating repeated measurements of an item. Each assessment timepoint is weighted in order to account for underlying linear trends in the data (i.e., the WEST-Trend), and to ensure that the rate of change (ROC) post-treatment is significantly greater than the null ROC expected at baseline (i.e., the WEST-ROC). Weighted scores for a given item are summed and analyzed using one-sample *t*-tests (one-tailed). Results were corrected for multiple comparisons using the Bonferroni procedure (i.e., alpha was set at 0.05/4 = 0.013). For detailed descriptions of this analysis approach, please see Simic et al. (23) for a comparable study design using PCA therapy, and Howard et al. (82). Following Howard et al.'s (82) recommendations, treatment effects were considered significant only when both the WEST-Trend and WEST-ROC analyses showed significant results.

Due to the small sample size, changes in performance on the WAB, BNT, CETI and CCRSA (outcomes b, c, and d above) were analyzed using descriptive statistics. In addition, we evaluated whether changes on the WAB and BNT represented a clinically significant difference, according to published benchmarks (83). Similarly, descriptive statistics were used to track WM performance on digit and visual span tasks over time.

Descriptive statistics are also presented for the DCT (outcome e above), and conversational speech samples were analyzed using the Profile of Word Errors and Retrieval in Speech (POWERS) approach (84). Briefly, an independent rater blind to treatment condition and assessment time coded speech samples according to the following conversation parameters: substantive turns (i.e., a turn which contains at least one content word), minimal turns (i.e., a turn which does not contribute meaningfully to the conversation), content words (i.e., nouns, proper nouns, adjectives, adverbs, verbs and numerals), nouns (i.e., proper and common nouns), and word errors (i.e., circumlocutions, semantic paraphasias, phonological errors, neologisms, pauses greater than 2 s and filled pauses). Once coded, the following ratios were calculated (85): (a) minimal turns/total turns, (b) word errors/content words, (c) word errors/turns, (d) number of content words/substantive turns, and (e) nouns/substantive turns.

## Results

Six participants were recruited for the present study, and three each were randomly assigned to either the WMC or WMI conditions. Overall, participants had a mean age of 59.2 years (SD = 8.6 years), an average of 16 years of education (SD = 2.9 years), and were 2.83 years post-onset of stroke, on average (SD = 2.1). Individual participant details, as well as means for the WMC and WMI conditions can be seen in Table 1.

**Table 1:** Individual participant characteristics.

**Code**	**WM Condition**	**Sex**	**Age**	**Education (yrs)**	**Yrs Post Stroke**	**Aphasia Type**
WMI-1	Intervention	M	59	14	1.25	Anomic
WMI-2	Intervention	M	59	18	4	Anomic
WMI-3	Intervention	F	53	21	0.75	Conduction
WMC-1	Control	M	75	14	6	Broca's
WMC-2	Control	M	59	16	3.5	Anomic
WMC-3	Control	M	50	14	1	Broca's
	**Total Mean** (SD)	59.2 (8.6)	16 (2.9)	2.83 (2.1)	
	**WMI Mean** (SD)	57 (3.5)	17.7 (3.5)	2 (1.8)	
	**WMC Mean** (SD)	61.3 (12.7)	14.7 (1.2)	3.5 (2.5)	

*All participants had a single left-hemisphere cerebrovascular accident and were premorbidly right-handed*.

### Feasibility: Practicality and Acceptability

Overall, 37 participants introduced to the study from information sessions expressed an interest in participating. After an initial review of each participant's file, 25 did not meet inclusion criteria (e.g., due to a history of bilateral or right hemisphere strokes, no stroke etiology, or pre-existing psychological disorders). The remaining 12 were screened for eligibility. Of those, one presented with a pre-existing cognitive impairment, one with mild anomia, exceeding our cut-off of 75% naming accuracy on the BNT, and three with moderate-severe apraxia of speech (AOS). One participant also presented with AOS and comprehension difficulties and did not have computer nor Internet access. The resulting final sample size was six. Thus, our initial recruitment target of 20 participants (i.e., 10 in each condition) was not met.

However, the six participants who were enrolled in the study completed it, and feasibility metrics were excellent for protocol compliance, retention rate and lack of adverse events. All participants completed 100% of PCA therapy sessions, and 100% of WM training sessions. In addition, only one deviation from the protocol was noted: two participants in the WMC condition (WMC-1 and WMC-3) engaged in the active control task more than 5 days a week. Finally, with respect to the usability of the WM training task, results from the SUS indicate an average score of 69.2/100 for the WMI condition (i.e., WMI-1 = 65.0, WMI-2 = 57.5, and WMI-3 = 85.0), and an average score of 47.5/100 for the WMC condition (i.e., WMC-1 = 40.0, WMC-2 = 60.0, and WMC-3 = 42.5).

### Feasibility: Preliminary Efficacy and Communication Outcomes

#### Naming Accuracy of Treated and Untreated Words

Overall, all participants in both conditions showed improvements in naming accuracy for the treated words with a mean change of 45.6% (SD = 21.9%) from baseline- to post-treatment, and 42.2% (SD = 22.78%) from baseline- to 1-month follow-up. Individual participant WEST analyses indicate significantly improved naming accuracy in four participants (WMI-2, WMI-3, WMC-1 and WMC-3), two in each condition. Three of these participants showed significant improvements in naming accuracy at the 1-month follow-up stage as well (WMI-3, WMC-1 and WMC-3). For the untreated words, naming accuracy improved by a mean of 16.5% (SD = 11.7%) from baseline to post-treatment, and by a mean of 23.2% (SD = 14.8%) from baseline to 1-month follow-up. According to WEST analyses, two participants showed significant improvements in naming of the untreated words at post-treatment (WMC-2) and at 1-month follow-up (WMC-3), respectively (individual participant scores are presented in Table 2).

**Table 2:** Percent naming accuracy of treated and untreated words for each individual participant, across the three baseline periods, and at post-treatment and follow-up.

	**B1**	**B2**	**B3**	**Mean (B1–B3)**	**Post**	**4WFU**
**Treated words (%)**							
Intervention	WMI-1	0.00	6.67	16.67	7.78	26.67	30.00
	WMI-2	16.67	6.67	20.00	14.44	66.67*	46.67
	WMI-3	10.00	10.00	16.67	12.22	86.67*	90.00*
Control	WMC-1	10.00	26.67	6.67	14.44	80.00*	76.67*
	WMC-2	33.33	13.33	3.33	16.67	46.67	53.33
	WMC-3	3.33	0.00	0.00	1.11	33.33*	23.33*
	**Mean**	**12.22**	**10.56**	**10.56**	**11.11**	**56.67**	**53.33**
	*SD*	*11.86*	*9.05*	*8.28*	*9.73*	*24.86*	*25.99*
**Untreated Words (%)**							
Intervention	WMI-1	3.33	0.00	10.00	4.44	10.00	13.33
	WMI-2	16.67	6.67	13.33	12.22	16.67	30.00
	WMI-3	0.00	6.67	26.67	11.11	30.00	60.00
Control	WMC-1	13.33	16.67	6.67	12.22	30.00	23.33
	WMC-2	20.00	3.33	26.67	16.67	53.33*	46.67
	WMC-3	0.00	0.00	3.33	1.11	16.67	23.33*
	**Mean**	**8.89**	**5.56**	**14.44**	**9.63**	**26.11**	**32.78**
	*SD*	*8.86*	*6.21*	*10.04*	*8.37*	*15.55*	*17.31*

* *Significant WEST-ROC and WEST-Trend result, based on one-sample t-tests (one-tailed). Bonferroni-corrected for multiple comparisons (i.e., alpha = 0.05/4 = 0.013). Note. WEST weighting factors are based on three baseline measures and one post-therapy measure (i.e., either post-treatment, or 4r-week follow-up). B1, Baseline 1; B2, Baseline 2; B3, Baseline 3; 4WFU, 4-week follow-up*.

#### Aphasia and Anomia Severity

Individual participant WAB-AQ and BNT scores at each assessment time are presented in Table 3. Individuals in the WMI condition showed an average improvement on the WAB-AQ of 5.4 points pre- to post-treatment, and of 8.6 points from pre- to 1-month follow-up. This change indicates a clinically significant difference (i.e., a change of greater than five points, or 5%). In comparison, individuals in the WMC condition did not attain this benchmark, showing an average WAB-AQ improvement of 3.7 points pre- to post-treatment, and 3.8 points pre- to 1-month follow-up. It is important to note, however, that individuals in the WMC condition presented with overall lower WAB-AQ scores.

**Table 3:** Individual participant scores on measures of anomia and aphasia severity, communicative effectiveness and confidence, WM and discourse comprehension across assessment times.

	**WM Intervention**				**WM Control**			
	**WMI-1**	**WMI-2**	**WMI-3**	***Mean***	**WMC-1**	**WMC-2**	**WMC-3**	***Mean***
**Western Aphasia Battery-Revised-Aphasia Quotient (WAB-R-AQ)**								
Pre	76.8	81.1	79.4	*79.1*	66.2	77.6	43.6	*62.5*
Post	85.2	82.5	85.7	*84.5*	72.2	77.2	49.0	*66.1*
1-month	84.6	92.9	85.7	*87.7*	76.6	81.3	40.9	*66.3*
**Boston Naming Test (BNT)-Naming Accuracy (%)**								
Pre	13.3	38.3	40.0	*30.6*	65.0	55.0	15.0	*45.0*
Post	15.0	46.7	60.0	*40.6*	63.3	53.3	25.0	*47.2*
1-month	11.7	46.7	68.3	*42.2*	60.0	56.7	16.7	*44.4*
**Communication Effectiveness Index (CETI)-Patient (%)**								
Pre	52.4	82.4	46.4	*60.4*	47.5	65.3	54.3	*55.7*
Post	57.4	64.0	83.5	*68.3*	49.2	63.5	76.6	*63.1*
1-month	41.5	83.8	79.5	*68.3*	53.4	73.1	43.2	*56.5*
**Communication Effectiveness Index (CETI)-Partner (%)**								
Pre	47.8	71.0	61.6	*60.1*	46.1	60.9	77.6	*61.5*
Post	52.7	64.7	80.5	*66.0*	n/a*	n/a*	69.4	*-*
1-month	57.1	63.1	91.2	*70.5*	53.1	n/a*	71.3	*62.2*
**Communication Confidence Rating Scale for Aphasia (CCRSA; %)**								
Pre	57.5	72.5	65.0	*65.0*	60.0	75.0	60.0	*65.0*
Post	60.0	77.5	90.0	*75.8*	60.0	75.0	67.5	*67.5*
1-month	52.5	82.5	72.5	*69.2*	65.0	72.5	60.0	*65.8*
**Wechsler Memory Scale (WMS) Digit Span - Forward (max score** **=** **12)**								
Pre	8	7	0	*5.0*	4	8	0	*4.0*
Post	5	7	3	*5.0*	4	8	0	*4.0*
1-month	8	8	2	*6.0*	5	7	1	*4.3*
**Wechsler Memory Scale (WMS) Digit Span-Backward (max score** **=** **12)**								
Pre	4	3	2	*3.0*	2	3	1	*2.0*
Post	5	3	4	*4.0*	3	3	0	*2.0*
1-month	4	3	4	*3.7*	2	4	3	*3.0*
**Corsi Block Tapping Visual Span-Forward (max score** **=** **14)**								
Pre	10	5	8	*7.7*	6	5	5	*5.3*
Post	9	7	8	*8.0*	7	9	7	*7.7*
1-month	11	7	9	*9.0*	6	10	4	*6.7*
**Corsi Block Tapping Visual Span-Backward (max score** **=** **12)**								
Pre	7	7	6	*6.7*	7	7	7	*7.0*
Post	8	5	9	*7.3*	6	7	6	*6.3*
1-month	8	6	10	*8.0*	10	8	6	*8.0*
**Discourse Comprehension Test (DCT)-Auditory (%)**								
Pre	92.5	92.5	77.5	*87.5*	75.0	77.5	65.0	*72.5*
Post	70.0	95.0	82.5	*82.5*	82.5	90.0	60.0	*77.5*
1-month	90.0	90.0	87.5	*89.2*	72.5	75.0	52.5	*66.7*

* *CETI score not available (not completed accurately)*.

With respect to anomia severity, participants in the WMI condition demonstrated an average of 10% improvement in naming accuracy on the BNT pre- to post-treatment, and 11.7% pre- to 1-month follow-up. This corresponds to an improvement in naming on six and seven items on the BNT, respectively. As with the WAB-AQ, a change of greater than three points out of 60 (or 5%) indicates a clinically significant difference. In comparison, individuals in the WMC condition showed an improvement in BNT naming accuracy of 2.2% pre- to post-treatment, and a slight decrease (0.6%) pre- to 1-month follow-up.

#### Communication Effectiveness and Confidence

According to self-ratings on the CETI, communicative effectiveness for participants in the WMI condition improved by an average of 7.9% pre- to post-treatment, and 7.8% pre- to 1-month follow-up. Participants in the WMC condition made a comparable improvement of 7.5% pre- to post-treatment, but communicative effectiveness largely returned to pre-treatment levels at the 1-month follow-up stage. According to partner ratings of communicative effectiveness, the participants in the WMI condition were rated 5.9% higher from pre to post treatment, and 10.4% higher from pre- to 1-month follow-up. Post-treatment CETI partner ratings were not available for two participants in the control condition (WMC-1 and WMC-2), and minimal (0.7%) change was seen in those in the WMC condition from pre- to 1-month follow-up.

Ratings of communication confidence based on the CCRSA show a similar pattern: participants in the WMI condition rated themselves 10.8% higher from pre- to post-treatment, and 4.2% higher pre- to 1-month follow-up. In comparison, those in the WMC condition rated themselves 2.5% higher pre- to post-treatment, which largely returned to pre-treatment levels at 1-month follow-up. Please see Table 3 for individual participant scores on the CETI and CCRSA.

#### WM Span Tasks

Although not the primary aim of the present study, results from WM tasks suggest impairments in WM digit span in our sample of participants with post-stroke aphasia. Overall better performance was observed in the visual span task for participants in both conditions. In addition, WMC-2 demonstrated improvements in the forward visual span task over time, whereas participants WMC-1 and WMI-3 demonstrated improvements in the backward visual span task over time. All other participants remained relatively stable in their performance on the WM span tasks. Please see Table 3 for details.

#### Discourse

Performance on the DCT did not notably change across assessment times (Table 3). Discourse analysis of conversational speech using the POWERS reveals variable performance across participants and assessment times (see Table 4). Smaller ratios of minimal turns/total turns, word errors/content words and word errors/turn indicate better performance (ratios 1–3 in Table 4). In the WMI condition, participant WMI-1 showed a decrease in these ratios pre- to post-treatment and maintained a small ratio of minimal turns/total turns at 1-month follow-up. Participants WMI-2 and WMI-3 demonstrated stable or increasing performance for these ratios. In the WMC condition, all participants show decreasing ratios of word errors/content words and word errors/turn across assessment times. The ratio of minimal turns/total turns remained stable or increased in the WMC condition, across assessment times.

**Table 4:** Conversational speech analysis using POWERS parameters and associated ratios across participants and assessment times.

	**Intervention**			**Control**		
	**WMI-1**	**WMI-2**	**MWI-3**	**WMC-1**	**WMC-2**	**WMC-3**
**1. Minimal Turns/Total Turns**						
Pre	0.27	0.24	0.19	0.4	0.13	0.44
Post	0.15	0.33	0.19	0.57	-	0.4
1-month	0.08	0.29	0.2	0.61	0.31	0.52
**2. Word Errors/Content Words**						
Pre	0.22	0.06	0.12	2	0.39	0.19
Post	0.05	0.17	0.26	1.47	-	0.07
1-month	0.23	0.15	0.28	0.89	0.28	0.1
**3. Word Errors/Turn**						
Pre	0.67	0.15	0.76	1.59	0.89	0.31
Post	0.2	0.49	3.33	0.49	-	0.08
1-month	1.34	0.44	4.48	0.21	0.52	0.12
**4. Number Content Words/Substantive Turns**						
Pre	4.31	4.19	8.2	1.97	3.2	3.22
Post	5.45	4.54	19.82	1.3	-	1.95
1-month	7.13	4.43	19.9	1.13	4.32	2.77
**5. Nouns/Substantive Turns**						
Pre	1.11	1.33	1.4	1.41	1.92	0.7
Post	1.26	0.98	2.24	0.87	-	0.64
1-month	2.06	1.62	4.1	0.92	1.26	1

*Post-treatment speech sample not available for WMC-2. All scores represent ratios based on POWERS conversational parameters [as per (84, 85)]*.

A larger ratio of content words/substantive turns, and nouns/substantive turns indicates better performance (ratios 4 and 5 in Table 4). In the WMI condition, participants WMI-1 and WMI-3 show increases in these ratios across assessment times; WMI-2 shows relatively stable performance across assessment times. In the WMC condition, minimal change and/or a slight decrease in these ratios is noted across assessment times.

### Interim Summary of Findings

The findings suggest trends toward greater improvement for individuals in the WMI condition (e.g., WAB-R AQ; BNT; patient reported CETI; CCSRA; conversational speech analysis). However, other results do not distinguish performance of individuals in the two conditions (e.g., performance on treated and untreated words; DCT). Since the number of individuals in each condition was too small to be analyzed separately as two conditions, we chose to compare in detail the performance of two well-matched patients from each condition, as described below.

### Case Comparison

Given the small number of participants, and the variability of findings summarized above, it is difficult to extract clear patterns in the data between the participants in the WMI and WMC conditions. Thus, in order to investigate the potential benefits of combining WM training with anomia therapy, we present here the case of participant WMI-3, who demonstrated a notable increase in WM training performance over the course of therapy (i.e., WMI-3 was performing the dual WM task at the 3-back level by the end of treatment). We compare the performance of WMI-3 to a participant in the control condition, WMC-2, who was well-matched in terms of age, education, years post-stroke (see Table 1), as well as pre-treatment WAB-AQ, naming performance and WM capacity (particularly for backward span tasks; see Tables 2, 3).

After therapy, WMI-3's naming accuracy for the treated words improved from 30 to 90% (compared to an increase from 30 to 50% for WMC-2). WEST analyses indicate significantly improved naming of the treated words post-treatment and at 1-month follow-up for WMI-3, but not for WMC-2. Interestingly, however, WMC-2 showed significant improvements in naming of the untreated words post-therapy.

In addition, WMI-3 demonstrated a mean increase of 7-points on the WAB-AQ (compared to a mean increase of 3-points for WMC-2). Similarly, mean percent change on the BNT was 24.2% for WMI-3, whereas WMC-2 showed no mean change on the BNT across assessment times. Interestingly, WMC-2 showed the greatest improvement on the forward visual span task over time (mean change of 32.2%), whereas WMI-3 showed the greatest improvement on the backward visual span task (mean change of 25.2%), which, much like the n-back task, places greater demands on WM updating capacity. Finally, WMI-3 but not WMC-2 showed improved ratings of communicative effectiveness, and communicative confidence.

On the DCT, WMI-3's performance improved by an average of 7.5% and demonstrated the greatest amount of improvement at 1-month follow-up. In comparison, WMC-2 showed an average improvement of 5% on the DCT, but this involved a return to baseline performance at 1-month follow-up. Conversational analyses using the POWERS indicate that although WMI-3 showed increased ratios of word errors/content words and word errors/turn, she also demonstrated improvements on the number of content words per substantive turn and nouns per substantive turn at post-treatment and at 1-month follow-up. In comparison, these same improvements were not observed for WMC-2 (see Table 4).

## Discussion

The aim of this pilot study was to evaluate the feasibility of a combined treatment approach which involved self-administered computerized adaptive dual n-back WM training, and clinician-administered PCA treatment for anomia in post-stroke aphasia. To assess feasibility, we also conducted an exploratory investigation into the preliminary efficacy of a combined therapy approach through detailed descriptions of trends in the data, as well as a case comparison.

### Feasibility: Practicality and Acceptability

Overall, our findings suggest that a self-paced, individualized WM program, in combination with targeted face-to-face naming therapy is feasible for individuals with post-stroke aphasia. Compliance and retention rates were excellent: participants followed through with both training protocols at the required schedule. Only a single protocol deviation was noted, whereby two participants in the WMC condition engaged with the active control task more than expected. However, there was no evidence that this influenced WM performance; the active control condition remained at the 1-back level, and as such did not increase in difficulty. The primary challenge in the present study was recruitment. Our initial recruitment target of 20 participants was not met, due to various barriers, including strict inclusion criteria and limited resources for face-to-face treatment. A potential solution to the latter may be to offer virtual (see (73)) and/or self-guided PCA treatment, which could access a greater number of individuals. The excellent compliance rates for the virtual WM training in this study suggest that virtually delivered therapy may indeed be an acceptable option for individuals with post-stroke aphasia. Although not eligible for other reasons, one participant who was screened for the study did not have computer or Internet access. Equipping individuals with the technology needed for virtual therapy, or connecting them with local telerehabilitation centers, may be necessary to remove accessibility barriers and implement virtual treatment approaches more broadly.

Results from the SUS indicate acceptable, but not excellent usability scores for the WMI condition. Usability ratings may have been impacted by difficulty with the n-back task (e.g., discriminating visual and auditory stimuli) or by the dual nature of the task, which may have been too challenging for some participants. A single n-back paradigm may be more appropriate for the post-stroke aphasia population, although this requires further investigation. It is important to continue to solicit feedback from individuals with aphasia, in order to gain valuable insights for additional aphasia-friendly modifications that can be made in future iterations of this WM training approach. As expected, those in the WMC condition found the active control task somewhat repetitive, which could explain the lower usability scores for the WMC condition. Despite these difficulties, all participants persisted and completed the WM training, which suggests that challenging tasks can be motivating in therapy.

### Feasibility: Preliminary Efficacy and Communication Outcomes

In the present study, preliminary efficacy was assessed using a variety of outcome measures, with a focus on exploring the added benefit in communication outcomes among individuals who received a combined WM training and anomia therapy approach (i.e., the WMI condition). Individual by-item analyses of naming accuracy for the treated and untreated words indicate significantly improved naming of the treated words for four participants overall—two in each condition. In addition, two participants demonstrated significant improvements in naming of the untreated words following therapy. These findings are in line with previous work demonstrating that PCA induces significant changes in naming in approximately 70% of participants (19, 23). Although the small sample precluded statistical analysis, naming improvements were also noted on the BNT (i.e., an untrained naming task). Interestingly, participants in the WMI condition made clinically significant improvements on the BNT, whereas those in the WMC condition demonstrated limited change.

This pattern is echoed in the participants' WAB-R performance over time. While participants in both the WMI and WMC conditions demonstrated improvements in the WAB-R AQ following intervention, only those in the WMI condition demonstrated a clinically significant change of greater than 5 points (83). These results suggest a trend toward treatment-induced changes in anomia and aphasia severity (i.e., becoming milder) for those in the WMI condition. The comparison of two well-matched cases provides further support for this trend. Participants WMI-3 and WMC-2 presented with comparable demographics, naming performance and aphasia severity (i.e., WAB-R AQ) prior to treatment. Importantly, they also demonstrated comparable pre-treatment WM capacity. Participant WMI-3 showed great improvements during WM training. Following therapy, WMI-3 demonstrated significant improvements in naming accuracy for the treated words, and clinically meaningful improvement on the BNT and WAB-AQ. WMC-2 did not show improvements in these areas. A similar pattern was seen when comparing WMI-3 and WMC-2 on communicative effectiveness, and communicative confidence. In fact, participants in the WMI condition showed overall greater and/or longer-lasting improvements in ratings of communicative effectiveness and confidence, compared to those in the WMC condition.

Although not a primary aim of this study, we also tracked changes in WM performance over time, with a particular interest in performance on the backward visual and digit span tasks, which are more closely associated with WM (as opposed to STM) capacity. Of note, all participants demonstrated some WM deficits on the digit span task, corroborating previous work (36, 40–43). Performance on the visual span task was generally better than performance on the digit span task, suggesting that the former may be a more appropriate measure of WM in individuals with aphasia, as it may remove some of the confounds associated with the language impairments in question. No discernible differences in WM performance were noted between individuals in the WMI and WMC conditions.

Interestingly however, participant WMI-3, who showed great progress during WM training, also demonstrated a large improvement in performance on the backward visual span task (i.e., a measure of WM that more closely resembles the updating demands of the n-back task). In comparison, WMC-2, who was in the active control condition, demonstrated a large improvement in performance on the forward visual span task (i.e., a measure of STM). In line with previous work [e.g., (54)], this finding encourages more research on the potential for WM training programs (such as *N-Igma*) to improve WM capacity in people with aphasia.

Finally, participants in both conditions demonstrated some improvement in their conversational discourse, tending to contribute more meaningfully and accurately to conversations following therapy (i.e., reducing minimal turns and word errors). However, participants in the WMI condition also demonstrated an increased proportion of content words and nouns following therapy. As above, this is underlined in the comparison of WMI-3 and WMC-2, whereby the former (but not the latter) demonstrated improvements in the proportion of content words and nouns used in conversational speech. This finding may suggest that treatment at the single-word level can transfer to a discourse task, possibly by increasing the availability and use of content words and nouns. The added benefit of WM training may be to support the active maintenance of, and access to, the words needed in a conversational task, as has been suggested in previous work (45).

Although improvements were noted in discourse production, limited change was observed in discourse comprehension on the DCT. This result may be due to higher levels of comprehension at baseline across the participants, or otherwise, because the DCT is not an appropriate outcome measure for the PCA treatment approach (i.e., which primarily targets production abilities). Previous work has shown that WM training in individuals with aphasia can lead to improvements in sentence comprehension [e.g., (54)]. Thus, perhaps a sentence (rather than discourse) comprehension task may be a more appropriate outcome measure to include in future studies.

### Limitations

The primary limitation of the present study is the small sample size, which only allowed for a descriptive analysis of the data. Given the numerous factors that can impact treatment outcomes in individuals with post-stroke aphasia, replication in a larger and more homogeneous sample is imperative. Also, it is notable that while participants in the WMI condition presented with somewhat milder aphasia (based on the WAB-R AQ), they also presented with a more severe anomia (based on the BNT). As such, pre-therapy status between individuals in the intervention and control conditions was not matched which may have influenced the results.

## Conclusion

Further research is needed to better understand the efficacy of combined WM training and language therapy for the treatment of aphasia (54). As well, further investigation of the different cognitive abilities that potentially underpin treatment response at different stages of recovery is warranted (45). Nevertheless, the present findings suggest that a combined treatment for both WM and naming deficits in individuals with post-stroke aphasia is not only feasible but may have the potential to augment treatment efficacy and support generalization to broader communication contexts.

## Data Availability Statement

The original contributions presented in the study are included in the article, further inquiries can be directed to the corresponding author.

## Ethics Statement

The studies involving human participants were reviewed and approved by the University of Toronto Research Ethics Board (#25523), Aphasia Institute (no REB #), March of Dimes Aphasia and Communication Disabilities Program (no REB #). The patients/participants provided their written informed consent to participate in this study.

## Author Contributions

ER, CL, GE, and SB contributed to conception and design of the study. LL managed patient recruitment and data collection. NB, KM, and J-LT performed discourse analyses. TS analyzed and interpreted data and wrote the manuscript. ER, CL, GE, and LL contributed to manuscript writing and revision. All authors approved the submitted version.

## Funding

This work was supported by a catalyst grant from the Heart and Stroke Foundation Canadian Partnership for Stroke Recovery (CPSR), awarded to ER, CL, GE, and SB.

## Conflict of Interest

GE has a patent pending involving N-Igma. The remaining authors declare that the research was conducted in the absence of any commercial or financial relationships that could be construed as a potential conflict of interest.

## Publisher's Note

All claims expressed in this article are solely those of the authors and do not necessarily represent those of their affiliated organizations, or those of the publisher, the editors and the reviewers. Any product that may be evaluated in this article, or claim that may be made by its manufacturer, is not guaranteed or endorsed by the publisher.
